# Presumed Primary Bacterial Rhinosinusitis-Associated Optic Neuritis in a Cat

**DOI:** 10.3389/fvets.2020.00122

**Published:** 2020-03-12

**Authors:** Rachael Moghaddam, Jared A. Jaffey, Eric T. Hostnik, Alexandra Brower, Kathryn L. Wycislo

**Affiliations:** ^1^Department of Specialty Medicine, College of Veterinary Medicine, Midwestern University, Glendale, AZ, United States; ^2^Department of Veterinary Clinical Sciences, College of Veterinary Medicine, The Ohio State University, Columbus, OH, United States; ^3^Department of Pathology and Population Medicine, College of Veterinary Medicine, Midwestern University, Glendale, AZ, United States

**Keywords:** *Escherichia coli*, *Actinomyces* spp., epistaxis, feline, rhinitis, optic neuritis, rhinosinusitis

## Abstract

Acute bacterial rhinosinusitis is a common illness in children and can lead to complications such as preseptal/orbital cellulitis, orbital/subdural/cerebral abscessation, osteomyelitis, meningitis, and optic neuritis with blindness. Primary bacterial infections leading to rhinosinusitis in cats is rare and descriptive reports are lacking. The current report describes a cat with *Escherichia coli* and *Actinomyces* spp. infections causing severe chronic rhinosinusitis and subsequent loss of vision. Treatment with antibiotics and prednisolone coincided with a complete resolution of nasal disease-related clinical signs and substantial improvement in vision. This is the first description of a cat with presumed severe primary bacterial rhinosinusitis resulting in optic neuritis and loss of vision.

## Background

Chronic nasal disease is a commonly encountered disorder in cats that continues to challenge and frustrate veterinary clinicians. Chronic rhinosinusitis (CRS) is the second leading cause of chronic nasal discharge and sneezing in cats (1–5). The specific etiology of CRS in cats remains unknown but primary bacterial infections are considered rare (1–6). Acute bacterial rhinosinusitis (ABS) is a common illness in children, and depending on the location of the infection, can result in serious orbital and intracranial complications including preseptal/orbital cellulitis, orbital/subdural/cerebral abscessation, osteomyelitis, meningitis, and optic neuritis with blindness (7, 8). Delay in treatment of ABS in children can result in rapid progression of infection leading to irreversible visual impairment or life-threatening intracranial complications (9). The paucity of literature describing primary bacterial CRS in cats can make a timely diagnosis difficult, which can have devastating consequences. This report aims to provide a thorough description of clinical features, diagnostic findings, advanced imaging, and therapeutic interventions in a cat with a concurrent *Escherichia coli* and *Actinomyces* spp. infection causing severe CRS and loss of vision.

## Case Presentation

A 7-year-old neutered male Domestic Shorthair cat weighing 3.71 kg (8.2 lb), was presented to the Companion Animal Clinic at Midwestern University College of Veterinary Medicine (MWU-CVM) (day 1) with a 4-month history of sneezing, intermittent bilateral epistaxis and mucopurulent nasal discharge, progressive vision loss, as well as a 24-h onset of difficulty breathing, anorexia, and marked weakness. The cat was housed indoors only and had no contact with other animals. There was no known trauma or access to rodenticide. Two days before presentation, the primary care veterinarian performed coagulation testing (PT and PTT), serum biochemistry, and urinalysis, which were unremarkable. A complete blood count performed at that time revealed thrombocytopenia (23 × 10^3^/μL; reference interval 151–600 × 10^3^/μL) and a hematocrit within the reference interval (39%; reference interval 30–52%). A slide review with manual platelet count was not performed. In addition, the cat tested negative for feline leukemia virus (FeLV) antigen and feline immunodeficiency virus (FIV) antibody (IDEXX Laboratories, Inc., Westbrook, ME, USA). The cat also had a mean indirect systolic blood pressure measurement performed by Doppler ultrasonography that was unremarkable (149 mmHg; reference <160 mmHg).

Pertinent physical examination findings on day 1 included a rectal temperature of 103.5°F (39.7°C), heart rate of 220 beats per minute with a grade 2/6 systolic left parasternal heart murmur, respiratory rate of 35 breaths per minute with open mouth breathing as well as stertor and pale- pink oral mucous membranes. Ophthalmic examination performed by a boarded-small animal internist revealed bilaterally mydriatic pupils, intact pupillary light and palpebral reflexes, and an absent menace response. There was no evidence of intraocular or ocular surface inflammation. Intraocular pressure measurements performed with a tonometer (Icare TONOVET tonometer TV01, Vantaa, Finland) were unremarkable (OD: 15 mmHg, OS: 18 mmHg; reference range 10–25 mmHg). Indirect ophthalmoscopy was performed using a condensing lens and Finhoff transilluminator and revealed bilaterally swollen optic discs and prominent retinal vessels; there was no evidence of retinal detachment. There was a moderate amount of serosanguinous to mucoid nasal discharge and absent airflow bilaterally. The remainder of the physical examination revealed no additional abnormalities.

Clinically important complete blood cell count abnormalities performed at MWU-CVM were anemia (13%; reference interval 29–48%), reticulocytosis (81,200/μL; reference interval <45,000/μL), thrombocytopenia (57 × 10^3^/μL reference interval 200–500 × 10^3^/μL), leukocytosis (36.9 × 10^3^/μL; reference interval 3.5–16 × 10^3^/μL), neutrophilia (26.57 × 10^3^/μL; reference interval 2.5–8.5 × 10^3^/μL), with increased band neutrophils (1.1 × 10^3^ /μL; reference interval 0.0–0.15 × 10^3^/μL), monocytosis (5.2 × 10^3^/μL; reference interval 0.0–0.60 × 10^3^/μL), and total solids (5.8 g/dL; reference interval 5.2–8.8 g/dL). A blood smear reviewed by a boarded-clinical pathologist revealed an estimated manual platelet count of ~70 × 10^3^/μL and infectious organisms were not identified. A biochemical profile was not performed at that time.

Based on the age of the cat, chronicity of bilateral epistaxis that coincided with the development of progressive vision loss, absence of nasal airflow, and severe anemia, the cat was suspected to have an aggressive infiltrative nasal disease process with secondary blood loss anemia. Differential diagnoses considered most likely at that time included primary nasal neoplasia (e.g., adenocarcinoma, squamous cell carcinoma, lymphoma), abcessation, or fungal rhinitis. Initial treatment consisted of a blood transfusion to address the severe anemia. The cat was blood typed (type A) and was administered 9.4 mL/kg of type A packed red blood cell transfusion IV over 4 h. An hour after the completed blood transfusion, PCV and total solids increased to 21% and 6.4 g/dL, respectively.

**Figure 1:**
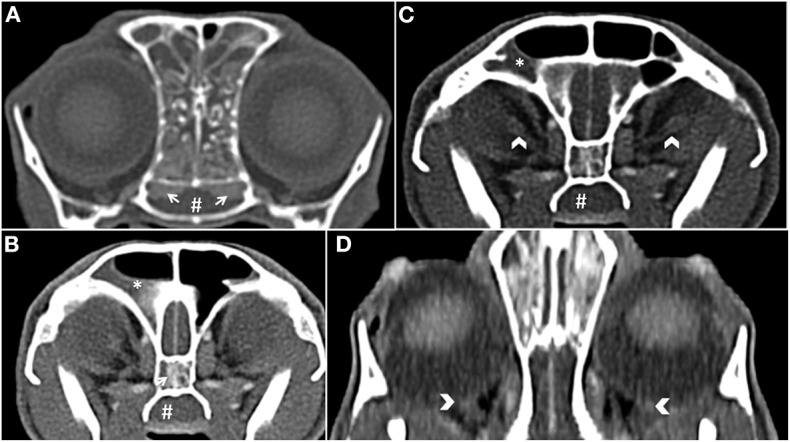
**(A)** Transverse plane post-contrast, white arrows show the contrast enhancement of the nasal mucosal tissue. There is also soft tissue abutting the osseous scrolls of the caudal nasal passage. The choanae are obscured by soft tissue (#) **(B)** Transverse plane post-contrast, there is dependent soft tissue within the right frontal sinus (*). There is also a white arrow showing enhancement of the mucosal tissue in the sphenoidal sinus and non-contrast enhancing tissue in the nasopharynx (#). **(C)** Transverse plane post-contrast, white chevrons show enhancing optic nerve bilaterally. **(D)** Dorsal oblique plane post-contrast, white chevrons highlight the optic discs.

Computed tomography (CT; CT scanner, Syngo VC 40 16-slice, Siemens Healthcare, Germany) imaging of the skull was performed on day 2. Helical images of the skull were acquired with a slice thickness of 1.00 mm and a pitch of 0.85. Soft tissue filled the majority of the nasal passages bilaterally, was non-contrast enhancing, and caused displacement of gas. The non-contrast enhancing soft tissue contoured to the shape of the turbinates. There was diffuse contrast enhancement of the soft tissue that lined the osseous scrolls (Figure 1A). The nasal soft tissue extended from the nares caudally to the ethmoid turbinates and into the frontal sinuses and sphenoidal sinus (Figure 1B). The soft tissue within the frontal sinuses collected in the dependent portion with fluid wicking along the margins (Figures 1B,C). The choanae were filled with soft tissue obstructing the patency of the nasopharyngeal upper airway (Figures 1A–C). There was no lysis of the paranasal bones or nasal septum. There was focal enhancement within the posterior ocular globes within the region of the optic disc and the optic nerve heterogeneously contrast enhanced (Figures 1C,D). Rigid rhinoscopy (Karl Storz multi-purpose rigid scope 30°, Segundo, CA, USA) was then performed, which demonstrated hyperemic, vascular, and edematous nasal mucosa with moderate hemorrhage and mucus in both nasal cavities. No obvious mass lesions or fungal plaques were identified. Copious amounts of mucopurulent material and blood were visualized in the nasopharynx using a flexible endoscope (Karl Storz Flexible Silver Scope Small Flexible Gastrointestinal scope, Segundo, CA, USA). Importantly, a nasal foreign body was not identified. Moist laparotomy sponges were placed in the caudal pharynx and 60 mL of chilled sterile 0.9% sodium chloride was used to flush each nasal cavity (120 mL total).

**Figure 2:**
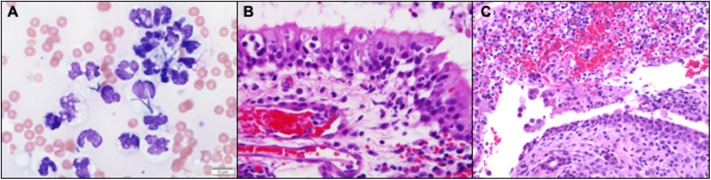
**(A)** Nasal biopsy impression smears revealed numerous markedly degenerate neutrophils with intracellular bacteria of variable, but predominantly rod-shaped, morphology (Wright-Giemsa, 100x). **(B)** Neutrophils extravasating from reactive and congested blood vessels and multifocally clustered within the respiratory mucosa. (Hematoxylin and Eosin stain). **(C)** Rounded, desquamated epithelium in a pool of suppurative exudate and hemorrhage that contains gram negative bacteria (Hematoxylin and Eosin stain).

Blind biopsies were obtained from each nasal cavity for cytology (tissue impression smear preparations), histopathology, and aerobic/anaerobic bacterial and fungal culture with antimicrobial susceptibility. Cytology results described the nasal samples as containing numerous degenerative neutrophils frequently containing intracellular bacterial rods of varying morphology with rare bacterial cocci noted (Figure 2A). Subsequent management consisted of ampicillin with sulbactam (30.5 mg/kg, IV, q 8 h), enrofloxacin (Baytril, Bayer HealthCare Animal Health, Shawnee Mission, KS, USA; 5.0 mg/kg, IV, q 24 h), Yunnan Baiyo (Yunnan Baiyao Group Corp. Ltd, Kunming, China; 1 capsule, PO, q 24 h), nebulization with 0.9% saline (q 6 h), dexamethasone-sodium phosphate (0.1 mg/kg IV, administered once), and Lactated Ringer's solution (5.4 mL/kg IV q 1 h) with 20 mEq/L potassium chloride.

The next day (day 3), the cat was no longer febrile 100.7°F (38.1°C), but a marked anemia returned with a PCV and total solids of 12% and 4.8 g/dL, respectively. The cat was administered 11.1 mL/kg of type A packed red blood cells over 4 h following identification of a compatible cross-match donor. An hour after the completed blood transfusion, the PCV and total solids increased to 21% and 6.4 g/dL, respectively. On the day of discharge (day 4), the cat was bright, alert, responsive, afebrile, and had a normal appetite. The anemia persisted but remained static 24 h after the blood transfusion (PCV: 22%; total solids: 6.8 g/dL). Medical management at the time of discharge included prednisolone (0.7 mg/kg, PO, q 24 h), Yunnan Baiyo (1 capsule, PO, q 24 h), pradofloxacin (Veraflox, Bayer HealthCare Animal Health, Shawnee Mission, KS, USA; 7.4 mg/kg, PO, q 24 h), and nebulization with 3 mls of 0.9% saline (q 12 h). Histopathologic sections revealed dense sheets of an almost pure population of neutrophils in which there were a myriad of bacterial colonies that appeared to have a short rod and thin filamentous morphology (Figures 2B,C). Aerobic/anaerobic bacterial culture results returned on day 6 and indicated the presence of *E. coli* and *Actinomyces* spp. (Supplemental Table 1) with an absence of fungal growth (confirmed on day 33). The cat was diagnosed with severe bacterial CRS and presumed optic neuritis associated loss of vision. Based on the antimicrobial susceptibility panel, the antimicrobial spectrum was expanded to include amoxicillin-clavulanic acid (Clavamox, Zoetis Inc., Kalamazoo, MI, USA; 16.9 mg/kg, PO, q 12 h) in addition the previously prescribed pradofloxacin (7.4 mg/kg, PO, q 24 h) on day 6.

The cat was presented on day 17 and had an improved appetite, energy, as well as decreased frequency of sneezing and epistaxis. Physical examination revealed unchanged ophthalmic abnormalities, absence of nasal discharge, and improved stertor. A complete blood count was performed and revealed resolution of anemia (HCT: 37%). Medical management with prednisolone, amoxicillin-clavulinic acid, pradofloxacin, Yunnan-Baiyo, and nebulization with 0.9% saline remained unchanged. By day 30 the owner reported that the sneezing persisted but had decreased in frequency and the epistaxis had ceased. The cat's vision was reported to be improved but not yet normal. Physical examination revealed a lack of nasal discharge and normal airflow bilaterally. Ophthalmic examination showed bilaterally mydriatic pupils, intact pupillary light reflexes, and a menace response could be elicited from both eyes. Indirect ophthalmoscopy was performed using a condensing lens and Finhoff transillumintor and revealed unremarkable optic discs and retinal vessels. Due to the improvement of clinical signs, the amoxicillin-clavulinic acid, pradofloxacin, and Yunnan Baiyo were discontinued. Therapy with prednisolone (0.7 mg/kg, PO, q 24 h) and nebulization with 0.9% sodium chloride (q 12 h) was continued.

On day 70, the cat was presented for evaluation and the owner reported complete resolution of sneezing, nasal discharge, and continued improvement of vision. The frequency of prednisolone administration was decreased and subsequently discontinued along with nebulization of saline. Follow-up with the owner by phone at the time of this writing (day 330) revealed that the cat had no sneezing, nasal discharge, and had improved, but not yet normal vision.

## Discussion

This report documents the clinical features, diagnostic findings, advanced imaging, and therapeutic interventions in a cat with presumed severe primary bacterial CRS and loss of vision caused by *E. coli* and *Actinomyces* spp. Chronic rhinosinusitis is a common cause of nasal discharge in cats second only to neoplasia. One small prospective study identified growth of mixed bacterial organisms from biopsy specimens in 60% (6/10) of cats with CRS (10). However, that study did not indicate if the cats with positive bacterial cultures were treated with antibiotics and if this resulted in complete resolution of clinical signs. This is important because it is difficult to determine whether bacteria cultured from the nasal passages are part of the normal nasal microbiota, primary pathogens, or are secondary to a primary etiology (3, 10). The absence of consistent follow-up data in observational studies that have identified bacterial culture positivity in cats with nasal disease limits our understanding of the frequency and clinical characteristics of primary pathogenic bacterial CRS. To the authors' knowledge, this is the first report of a cat with presumed primary bacterial CRS with extensive follow-up.

Evidence suggesting that *E. coli, Actinomyces* spp., or both were the primary cause of nasal disease in this cat includes: (a) there was a complete and sustained resolution of all clinical signs related to nasal disease subsequent to antibiotic administration and (b) the absence of sneezing or nasal discharge in the first 6.5 years of life. It is unclear as to the reason this cat developed bacterial CRS at 7 years of age. The cat had no clinical history of developing other persistent infections, making primary immunodeficiency unlikely. Secondary immunodeficiency cannot be ruled out, but is also unlikely due to the negative FeLV and FIV status. It remains a possibility that the cat had a foreign body that incited inflammation and was the source of bacterial infection. A non-dense object would have been difficult to identify with CT, especially among the copious soft tissue-attenuating mucoid material in the nasal cavity (11). Likewise, a foreign body would have been difficult to visualize with endoscopy because of the edematous tissue and thick mucoid material. Actinomyces infections have been reported to be associated with inhalation or ingestion of migrating grass awns and penetration of mucosa and soft tissue by inhaled foreign bodies (12).

Therapeutic antimicrobial use in companion animals has potential to promote the emergence of resistance in pathogenic as well as non-target commensal bacteria (13). Therefore, it is essential that antimicrobial therapy be aimed at known or most likely pathogens (13). The decision to initiate broad spectrum antimicrobial therapy in this cat with ampicillin/sublactam as well as enrofloxacin was made because there were multiple different types of potential pathogens (e.g., intracellular rods and cocci) identified on cytology of nasal tissue and the cat fulfilled the requisite criteria for a diagnosis of sepsis (14). De-escalation of antimicrobial therapy in the cat was attempted at the time of hospital discharge because he was stable. Pradofloxacin was chosen as the sole antibiotic at that time because it provides a broad spectrum of pathogen coverage involving both Gram-negative and Gram-positive aerobic and some anaerobic bacteria (15). In addition, pradofloxacin has a decreased resistance profile compared to other fluoroquinolones and would be expected to achieve ideal tissue penetration for the deep infection in the conchae (15). The bacterial culture and susceptibility diagnostic results returned on day 6 and indicated that the *E. coli* was susceptible to the fluoroquinolone. However, fluoroquinolones are not considered active against *Actinomyces* spp., the second cultured bacteria (16). The antimicrobial spectrum was then expanded to include amoxicillin-clavulanic acid and was chosen based on the susceptibility testing results. Both pathogens were susceptible to amoxicillin-clavulanic acid so the cat could have potentially been transitioned from pradofloxacin to only amoxicillin-clavulanic acid.

The cat in this report developed gradual loss of vision that coincided with the onset of sneezing, mucopurulent nasal discharge, and intermittent epistaxis. Rhinosinusitis in humans has been reported to lead to orbital complications such as optic neuritis and loss of vision. Vision loss in these cases have been proposed to result from four different pathophysiologic mechanisms including: (1) mechanical compression of the optic nerve (e.g., abscess or mucoceles), (2) direct extension of the infection to the optic nerve, (3) secondary to orbital inflammatory changes causing optic neuritis, and (4) venous congestion of the optic nerve due to thrombophlebitis and retinal artery occlusion due to increased pressures in the orbit (8, 17–20).

The specific cause for the development of optic neuritis and loss of vision in this cat is unknown but believed to be associated with direct extension of infection, inflammation, or both, into the orbits causing optic neuritis. The initial fundic examination revealed bilaterally swollen optic discs and prominent retinal vessels, likely a result of an inflammatory process. Further, the CT revealed abnormal contrast enhancement of the optic discs and optic nerve bilaterally. This assumption could further be supported by the gradual restoration of the cat's vision after treatment with antibiotics and steroids, decreasing the inflammation, and allowing the optic nerve to recover. A vascular etiology causing optic neuritis and loss of vision in this cat is possible but unlikely because of important anatomical differences between cats and people. The main supply of blood to the eye and orbit in cats is via the internal maxillary artery, which branches to give rise to the external ophthalmic artery. However, the overall blood supply to the cat eye is quite diverse. In comparison, the majority of orbital circulation in people is supplied via the internal ophthalmic artery, which traverses within the optic nerve. Inflammation or thromboses of the internal ophthalmic artery in people would likely affect both the optic nerve and vision, yet it would be unlikely for a single vascular event within the external ophthalmic artery in cats to have the same net result (21). A limitation of this report was the lack of magnetic resonance imaging, cerebrospinal fluid diagnostic evaluation, or electroretinography, which could have strengthened the supposition that the etiology of blindness was optic neuritis.

Whilst the cat's vision remained diminished, the owner reported a subjective improvement over time. The owner reported that the cat was able to play with toys, navigate through the house, and jump on surfaces though he occasionally missed the intended mark. Importantly, the cat's vision was not assessed objectively to definitively corroborate the owner's subjective evaluation of the cat's vision. It is unknown if the cat reported here will continue to regain vision over time. Improvement in vision of humans with rhinosinusitis associated optic neuritis following aggressive medical (i.e., antibiotics ± corticosteroids), surgical intervention, or both ranges from permanent blindness to restitution of normal vision (8). The cat in this report was treated with oral Yunnan Baiyo following acquisition of nasal biopsies in an effort to decrease hemorrhage. There are mixed results on the efficacy of Yunnan Baiyo in companion animals. One randomized, controlled, blinded study showed that dogs that had received Yunnan Baiyo the day before nasal biopsies experienced significantly shorter time to cessation of bleeding compared to control (22). Another study in dogs revealed that Yunnan Baiyo administration resulted in increased strength of clots measured by thromboelastography (23). However, other studies in apparently healthy dogs and cats highlighted that Yunnan Baiyo administration did not significantly alter thromboelastography parameters (24, 25). While the evidence supporting Yunnan Baiyo in the veterinary literature is mixed, the drug is well-tolerated with minimal reported adverse effects (24, 25).

## Conclusion

The cat presented in this report demonstrates that bacterial CRS should be considered in cats with severe nasal disease with and without loss of vision, irrespective of age. In addition, early recognition and therapeutic intervention are important in mitigating severe sequela of infection including, but not limited to, optic neuritis and vision loss.

## Data Availability Statement

The raw data supporting the conclusions of this article will be made available by the authors, without undue reservation, to any qualified researcher.

## Author Contributions

RM, JJ, EH, AB, and KW contributed management of case, collection of data, writing and editing manuscript, and review final submission.

### Conflict of Interest

The authors declare that the research was conducted in the absence of any commercial or financial relationships that could be construed as a potential conflict of interest.
