# Supplementary material for: Presumed Primary Bacterial Rhinosinusitis-Associated Optic Neuritis in a Cat
Source: Front Vet Sci. 2020 Mar 12;7:122. doi: 10.3389/fvets.2020.00122 (PMC7080855; doi:10.3389/fvets.2020.00122)
Supplement: Supplementary file 1 [file Table_1.DOCX]

Supplemental Table. Aerobic and anaerobic bacterial culture and sensitivity results obtained from nasal tissue of a cat diagnosed with presumed severe primary bacterial rhinosinusitis and secondary loss of visions due to optic neuritis.

|  | **Isolate organism** | |
| --- | --- | --- |
|  | *Escherichia coli* | *Actinomyces species* |
| **Antimicrobic** |  |  |
| Amikacin | ≤ 4 (S) | 32 (I) |
| Amoxicillin/Clavulanic Acid | 8 (S) | 8 (S) |
| Ampicillin | > 8 (R) | --- |
| Cefalexin | 4 (S) | --- |
| Cefazolin | 2 (S) | ≤ 2 (S) |
| Cefpodoxime | ≤ 1 (S) | --- |
| Ceftazidime | ≤ 4 (S) | --- |
| Cephalothin | --- | 4 (S) |
| Chloramphenicol | 8 (S) | ≤ 8 (S) |
| Doxycycline | 2 (S) | --- |
| Enrofloxacin | ≤ 0.12 (S) | --- |
| Erythromycin | --- | ≤ 0.25 (S) |
| Gentamicin | 0.5 (S) | 16 (R) |
| Imipenem | ≤ 1 (S) | 2 (S) |
| Marbofloxacin | ≤ 0.12 (S) | --- |
| Orbifloxacin | ≤ 1 (S) | --- |
| Piperacillin/Tazobactam | ≤ 8 (S) | --- |
| Tetracycline | ≤ 4 (S) | 1 (S) |
| Trimethoprim/Sulphamethoxazole | ≤ 0.5 (S) | > 4 (R) |
| Vancomycin | --- | ≤ 1 (S) |

S, susceptible; I, intermediate; R, resistant
